# Nuclear binding SET domain 1 alleviates cartilage ferroptosis in knee osteoarthritis by upregulating the krüppel‐like factor 9/autophagy‐related 14 pathway via H3K36me2 modification

**DOI:** 10.1002/ccs3.70027

**Published:** 2025-06-29

**Authors:** Qinglei Yang, Rugang Li, Zhiqiang Hu, Wengang Zhu, Hongying Yu

**Affiliations:** ^1^ Department of Arthropathy and Osteopathy Yuebei People's Hospital Affiliated to Shantou University Medical College Shaoguan Guangdong China; ^2^ Department of Nephrology Yuebei People's Hospital Affiliated to Shantou University Medical College Shaoguan Guangdong China; ^3^ Department of Pharmacy Yuebei People's Hospital Affiliated to Shantou University Medical College Shaoguan Guangdong China

**Keywords:** autophagy‐related 14, chondrocytes, ferroptosis, knee osteoarthritis, krüppel‐like factor 9

## Abstract

Knee osteoarthritis (KOA) is a progressive disease featured by cartilage damage. This study attempts to explore the role of nuclear binding SET domain 1 (NSD1) in KOA cartilage ferroptosis, thereby finding a new target for KOA treatment. Pathological changes, cartilage damage, and inflammatory cytokine levels in the established KOA mouse model were assessed. Primary mouse knee chondrocytes were separated, cultured, and challenged with IL‐1β to establish in vitro KOA models. Cell viability was determined, Reactive oxygen species levels and ferroptosis‐related factors were measured after interventions with NSD1, krüppel‐like factor 9 (KLF9), and acyl‐CoA synthetase long‐chain family member 4 (ATG14). Furthermore, the enrichment of NSD1 and H3K36me2 on the KLF9 promoter as well as the enrichment of KLF9 on the ATG14 promoter was analyzed. Binding site between KLF9 and ATG14 promoter was assessed. NSD1 was downregulated in KOA mouse cartilage tissues and IL‐1β‐challenged chondrocytes. KOA severity was alleviated, chondrocyte viability was promoted, and ferroptosis was quenched after NSD1 overexpression. NSD1 strengthened H3K36me2 to upregulate KLF9 expression, and KLF9 transcriptionally activated ATG14 expression. KLF9 or ATG14 knockdown could both partially reverse the protective role of NSD1 overexpression on KOA cartilage ferroptosis. NSD1 enhanced KLF9 expression to improve ATG14 expression via H3K36me2 modification, thus relieving KOA cartilage ferroptosis.

## Graphical abstract

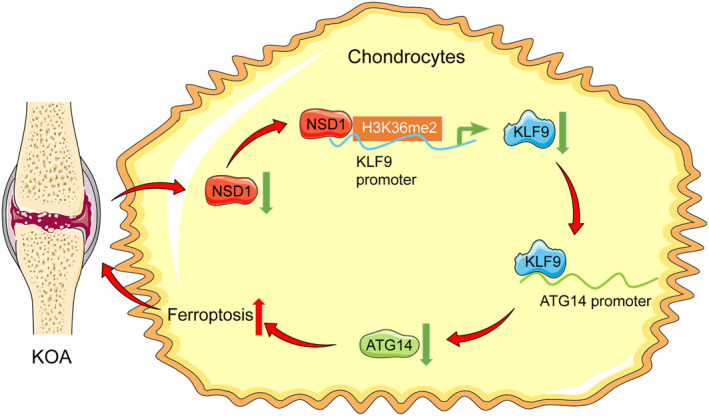

## 1 INTRODUCTION

As a heterogeneous disease that causes economic and clinical burdens around the whole world, osteoarthritis (OA) leads to a series of severe inflammatory responses in cartilage tissues and chondrocytes, thereby bringing about continuous pain, loss of mobility, and even disability to patients.^1^ Clinically speaking, the most common form of OA is knee OA (KOA).^2^ Nowadays, KOA is mainly triggered by increasing rates of aging and overweight.^3^ Drug usage, exercise, corticosteroid administration, and joint replacement are promising candidates for KOA treatment.^4^ Nevertheless, a practical and effective approach for KOA therapy remains to be probed.^5^ Ferroptosis is a crucial mechanism in KOA development as it could accelerate knee joint degeneration.^6^ Chondrocyte ferroptosis is necessarily related to oxidative stress injury and functional impairment in the KOA environment.^7^ Thus, in that sense, biomarkers targeting ferroptosis are an exciting prospect for KOA prevention.

Histone modification and RNA translation can accelerate KOA progression and disrupt therapeutic effects by influencing epigenetic regulation and gene integrity in patients.^8^ Histone H3 at lysine 36 (H3K36me) modification maintains gene homeostasis, transcription, construction, and DNA repair, and it is closely associated with human dysfunctional reactions.^9^ H3K36me is mainly regulated by nuclear binding SET domain 1 (NSD1) in different biological behaviors.^10^ Importantly, NSD1 affects H3K36me modification and quenches ferroptosis in KOA.^11^ It has been reported that NSD1 is inactivated in OA, which impacts cartilage structure, spoils chondrocyte differentiation, and decelerates metabolism,^12^ indicating the positive role of NSD1 in KOA treatment.

NSD1 could regulate transcriptional factors to be involved in chondrogenic differentiation and skeletal growth of fracture injury.^13^ Many transcriptional factors are actively involved in OA pathogenesis and development, and their protein levels are mediated by upstream genes, suggesting that transcriptional factors are attractive targets in KOA research.^14^ Among these transcriptional factors, krüppel‐like factor 9 (KLF9) is predominantly dysregulated in OA and is correlated with apoptosis and immune cell infiltration in OA patients.^15^ KLF9 interferes with chondrocyte proliferation, transformation, growth, damage, and death in OA.^16^ Functionally, KLF9 is capable of modulating levels of downstream genes through transcriptional promotion.^17^ Interestingly, acyl‐CoA synthetase long‐chain family member 4 (ATG14), a member of autophagic modulators, reverses autophagy deficiency, and promotes chondrocyte renewal in OA.^18^ ATG14 could also contribute to chondrocyte proliferation, mobility, viability, and migration, thus strengthening cartilage generation and development in KOA.^19^ Because of the above‐mentioned evidence pointing out the potential relation between these moleculars, we hypothesize that NSD1 might participate in KOA chondrocyte ferroptosis by promoting the KLF9/ATG14 pathway via H3K36me2 modification. Our findings may offer new therapeutic targets for KOA alleviation.

## 2 MATERIALS AND METHODS

### 2.1 Ethics statement

The protocol was also approved by the Institutional Animal Care and Use Committee of Yuebei People's Hospital Affiliated to Shantou University Medical College. Significant efforts were made to reduce the animal number and suffering.

### 2.2 Laboratory animals

A total of 48 healthy C57BL/6J male mice (8–10 weeks, 18–20 g, 20–23 g) (Shanghai SLAC laboratory animal Co., Ltd., Shanghai, China, SYXK (Shanghai) 2022‐0012) were allowed to acclimate for 1 week in a specific‐pathogen‐free environment with standard light and free access to food and water.

### 2.3 KOA model establishment and treatment

All laboratory mice (48) were randomly assigned into the sham group, KOA group, KOA + adenovirus (Ad)‐negative control (NC) group, and KOA + Ad‐NSD1 group (*n* = 6). In brief, anterior cruciate ligament transection (ACLT) surgery was performed to establish KOA mouse models. Mice were anesthetized with 2% sodium pentobarbital (2 mL/kg) via intraperitoneal injection, and the surgical area was routinely disinfected. A parapatellar incision was made on the medial side of the right knee joint, and the knee joint was fully flexed. The ACL and the anterior horn of the medial meniscus were exposed as much as possible during the surgery. The anterior horn was incised to remove the meniscus from the medial side. The ACL was severed under direct vision, and the anterior drawer test confirmed ACL rupture. The articular cartilage (AC) was protected during the surgery. The joint cavity was flushed with saline, and the joint capsule and skin were sutured layer by layer. Mice in the sham group were only subjected to skin incision without further intervention. The joint cavity of mice in the KOA group was injected with 1 × 10^9^ pfu (8 μL) of Ad‐NSD1 (Ad‐packaged NSD1 overexpression vector) or Ad‐NC (Ad‐packaged empty vector) 24 h prior to surgery. Ad vectors were designed and packaged by Hanbio Biotechnology (Shanghai, China). Mice were euthanized by intraperitoneal injection of sodium pentobarbital (200 mg/kg) 8 weeks after surgery.^11^ After euthanasia, mice were placed in the supine position, the forelimbs were immobilized, the skin and soft tissues of the hind limbs were excised to expose the knee joints, and the AC tissues were removed for subsequent analysis. In each group, 6 mice were randomly selected for histopathological observation and the other ones were homogenated for further experiments.

### 2.4 Senna O and fast green staining

Senna O staining was performed to assess cartilage tissue damage. In short, AC tissue sections were stained with 0.2% fast green solution (C500016‐0500), 1% acetic acid solution (A501931‐0005) and 0.1% Senna O solution (A600815‐0025) (all from Sangon Biotech, Shanghai, China). The sections were then dehydrated, cleaned, and fixed with neutral balsam (Sangon Biotech). Cartilage degeneration was assessed by three independent investigators according to the Osteoarthritis Research Society International (OARSI) grading method.^20^ The grading was classified from 0 to 6. Grade 0: intact cartilage and surface; grade 1: intact surface; grade 2: surface incontinuity; grade 3: vertical fracture; grade 4: erosion; grade 5: denudation; grade 6: deformation.

### 2.5 Enzyme‐linked immunosorbent assay

The levels of tumor necrosis factor‐α (TNF‐α) (ab108910, Abcam Inc., Cambridge, MA, USA), interleukin (IL)‐6 (ab100713, Abcam), and IL‐10 (E‐EL‐M0046, Elabscience Biotechnology, Wuhan, Hubei, China) in cartilage tissues of mice were analyzed using the specific enzyme‐linked immunosorbent assay (ELISA) kit according to the instructions.

### 2.6 Cell separation, cultivation, and treatment

Primary mouse chondrocytes were separated from knee cartilage of 5‐day male C57BL/6J mice. Cartilage tissues were dissected into fragments and digested with 2.5 mg/mL type II collagenase (17,101,015, Gibco Company, Carlsbad, CA, USA) for 2 h at 37°C and then digested with 0.5 mg/mL type II collagenase overnight. Primary chondrocytes were resuspended and cultured in Dulbecco’s modified Eagle medium (DMEM) (Gibco) containing 10% fetal bovine serum (A5670701, Gibco) and 1% penicillin‐streptomycin at 37°C in an incubator with 5% CO_2_. When cells reached about 70% confluence, exogenous nucleic acids were transiently transfected with Lipofectamine 3000 transfection reagent (L3000015, Thermo Fisher, Waltham, MA, USA) according to the manufacturer's instructions. oe‐NSD1, oe‐KLF9, and oe‐NC were purchased from Sangon Biotech, and short hairpin (sh)‐KLF9, sh‐ATG14, and sh‐NC from Hanbio Biotechnology. After 48 h of transfection, cells were harvested for total protein/RNA separation or treatment with 10 ng/mL IL‐1β^21^ for 24 h to establish in vitro KOA models, with untreated chondrocytes as the NC. NSD‐inhibitor (IN)‐2 (0.9 μM, MedChemExpress LLC, NJ, USA) was supplemented into chondrocytes,with the same amount of dimethylsulfoxide as the control, and then expression of relevant genes were verified.

### 2.7 CCK‐8 method

Cells (1 × 10^4^) were seeded into 96‐well plates and cultured with 10 μL cell counting kit‐8 (CCK‐8) solution (Dojindo Laboratories, Mashiki‐machi, Kumamoto, Japan) at 37°C for 4 h. Subsequently, the optical density value at 450 nm was determined with a microplate reader (BioRad Laboratories Inc., Hercules, CA, USA).

### 2.8 ROS assessment

Reactive oxygen species (ROS) level in cells was assessed using ROS kits (S0033, Beyotime Biotechnology, Shanghai, China). Briefly, cells were cultured with 10 μM dichloro‐dihydro‐fluorescein diacetate for 20 min at 37°C before 2 washes with PBS. Subsequently, fluorescence intensity was measured using a fluorescence microplate reader (Glomax Explorer, Promega Corporation, Milan, Italy). ROS kit (BB‐470538, Bestbio Biotechnology, Shanghai, China) was utilized to detect ROS levels in fresh cartilage tissues in compliance with the manufacturer's instructions.

### 2.9 Detection of Fe^2+^, MDA and GSH levels

Fe^2+^ level in tissue homogenates and cells was detected using an Iron Assay kit (ab83366), while malonaldehyde (MDA) level was detected using Lipid Peroxidation Assay kit (ab118970), and glutathione (GSH) level was measured using GSH/GSSG Ratio Detection Assay kit (ab138881) (all from Abcam) under the instructions.

### 2.10 Quantitative real‐time polymerase chain reaction

Trizol Reagent (Invitrogen, Carlsbad, CA, USA) was appointed to extract total RNA from cartilage tissues and chondrocytes, and the concentration of extracted RNA was assessed. Afterward, 2 μg total RNA was employed to synthesize cDNA using BeyoRT™ III cDNA First Strand cDNA Synthesis kit (D7178L, Beyotime). Quantitative real‐time polymerase chain reaction (qRT‐PCR) was carried out using BeyoFast™ SYBR Green qPCR Mix (D7260, Beyotime). With glyceraldehyde‐3‐phosphate dehydrogenase as an internal reference gene, relative expression levels were calculated with the 2^−ΔΔCt^ method. Primer sequences are listed in Table 1.

**TABLE 1:** Primer sequence of qRT‐PCR.

	Forward primer (5′‐3′)	Reverse primer (5′‐3′)
NSD1	ATTTGGGCAAAATTCAAGAGACG	GCCTCCTATTGGCAACTTTCATT
KLF9	ACCTTCGAGGGGTCACGATA	CGGCTTCTACCCTCAAAGCA
ATG14	ACACTCAGAGGCACCACATG	GTTGCAGCTTTCCCAATGCA
FTH1	CTGGAACTGCACAAACTGGC	CTCTCATCACCGTGTCCCAG
SLC7A11	CCCGATCTTTGTTGCCCTCT	AGGTCTCCGGAGAAGAGCAT
GPX4	ATGAAAGTCCAGCCCAAGGG	GCTAGAGATAGCACGGCAGG
ACSL4	CTCACCATTATATTGCTGCCTGT	TCTCTTTGCCATAGCGTTTTTCT
KLF9 promoter	AGAGGAAGGAAAGGCTTGCC	CTTGGGCGCCTTTGACTCTA
ATG14 promoter	CCCGCACCTTCACTGAGAAT	GAGCTCAGAGGCGGAGAATC
GAPDH	AGGTCGGTGTGAACGGATTTG	TGTAGACCATGTAGTTGAGGTCA

Abbreviations: ACSL4, acyl‐CoA synthetase long‐chain family member 4; ATG14, autophagy‐related 14; FTH1, ferritin heavy chain 1; GAPDH, glyceraldehyde‐3‐phosphate dehydrogenase; GPX4, glutathione peroxidase 4; KLF9, krüppel‐like factor 9; NSD1, nuclear binding SET domain 1; qRT‐PCR, quantitative real‐time polymerase chain reaction; SLC7A11, solute carrier family 7 member 11.

### 2.11 Western blot analysis

Total protein was extracted with radio‐immunoprecipitation assay lysis buffer (P0013B, Beyotime), and the concentration of total protein was analyzed using BCA kits (P0011, Beyotime). Subsequently, 30 μg total proteins were separated by sodium dodecyl sulfate‐polyacrylamide gel electrophoresis and then transferred onto polyvinylidene fluoride membranes, which were then sealed with 5% skim milk and incubated with primary antibodies against NSD1 (1:1000, PA5‐50857, Invitrogen), KLF9 (1:500, 701,888, Invitrogen), acyl‐CoA synthetase long‐chain family member 4 (ACSL4, 1:10,000, ab155282, Abcam), ferritin heavy chain 1 (FTH1, 1:1000, ab183781, Abcam), solute carrier family 7 member 11 (SLC7A11, 1:1000, ab307601, Abcam), GSH peroxidase 4 (GPX4, 1:2000, ab125066, Abcam), ATG14 (1:1000, ab315009, Abcam), and β‐actin (1:2000, ab8227, 2000) at 4°C overnight. After washing, the membranes were incubated with a secondary antibody immunoglobulin G (IgG, 1:5000, ab205718, Abcam). Protein expression was determined with enhanced chemiluminescence assay reagent (P0018S, Beyotime), with β‐actin as an internal reference, and the comparison data of the gray values between target protein and β‐actin were collected to calculate the relative expression level.

### 2.12 ChIP assay

Chromatin immunoprecipitation (ChIP) assay was performed under the instructions of SimpleChIP Plus Enzymatic Chromatin IP Kit (9005S, CST, Cell Signaling Technology). The samples were cultured with 1% formaldehyde at room temperature for 10 min to cross‐link protein and DNA, and then glycine was added to quench the reaction for 5 min. DNA fragmentation was performed according to the kit's instructions. Probe detection was performed on immunoprecipitated chromatin complexes using anti‐NSD1 (1:50, PA5‐50857, Invitrogen), H3K36me2 (1:30, ab176921, Abcam), KLF9 (1:30, 701,888, Invitrogen) and NC‐IgG (1:50, ab172730, Abcam). DNA was extracted after the decrosslinking between NaCl and proteinase K. QRT‐PCR was performed to detect the enrichment levels of NSD1 and H3K36me2 on the KLF9 promoter as well as KLF9 enrichment levels on the ATG14 promoter. Primer sequences are listed in Table 1.

### 2.13 Dual‐luciferase reporter gene assay

The synthesized ATG14‐wild type (WT) or ATG14‐mutant type (MUT) containing binding sites with KLF9 was transferred into pCMV‐report plasmid (16,156, Thermo Fisher), and cells were co‐transfected with oe‐NC or oe‐KLF9. Eventually, 48 h after transfection, cells were collected and lysed. Luciferase activity was determined using a dual‐luciferase assay kit (K801‐200, Biovision, Mountain View, CA, USA).

### 2.14 Statistical analysis

SPSS 21.0 (IBM SPSS Statistics) was applied for data analysis and GraphPad Prism 8.0 software (GraphPad Software Inc.) was employed for graphing. The results were exhibited as mean ± standard deviation. All data were inspected with normality distribution and homogeneity of variance test. Comparisons between two groups were analyzed with *t* test, and comparisons among multiple groups were analyzed with one‐way or two‐way analysis of variance (ANOVA). Tukey's multiple comparisons test was used for a post hoc test. The *p* value was obtained by a two‐tailed test, with *p* < 0.05 indicating statistical differences and *p* < 0.01 indicating extremely significant differences.

## 3 RESULTS

### 3.1 NSD1 overexpression alleviates cartilage damage in KOA mice

**FIGURE 1:**
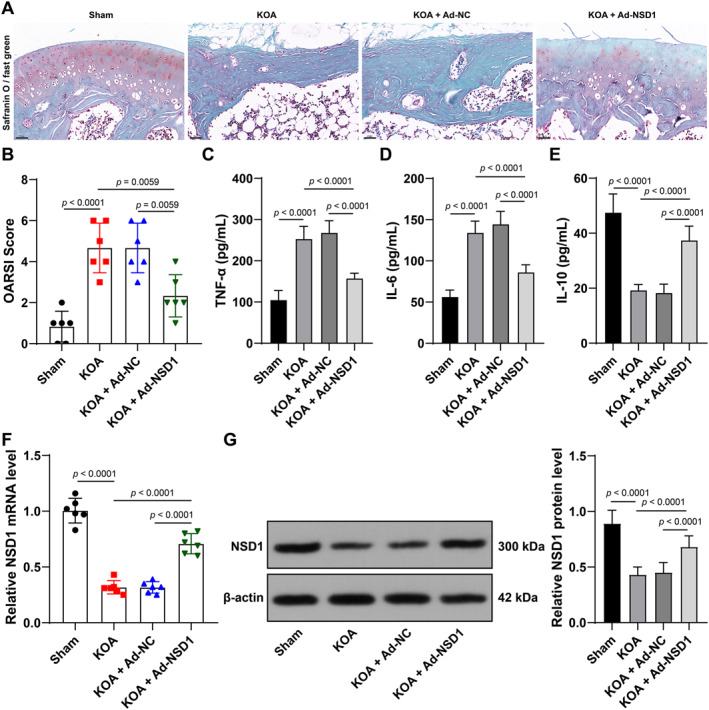
NSD1 overexpression alleviates KOA mouse cartilage damage. Ad‐NSD1 was injected into mice to upregulate NSD1 expression, with Ad‐NC as a control. KOA mouse model was established 24 h after injection and mice were euthanatized, and their knee joints were collected. (A) tissue pathological changes observed by senna O and fast green staining. (B) cartilage damage analyzed by OARSI scoring. (C–E) levels of tumor necrosis factor‐α, IL‐6, and IL‐10 in cartilage tissues determined by enzyme‐linked immunosorbent assay. (F, G) NSD1 expression in cartilage tissues assessed by quantitative real‐time polymerase chain reaction (F) and western blot analysis (G). *N* = 6. Data were presented as mean ± standard deviation. One‐way analysis of variance was used to analyze data in panels (B–G). Tukey's multiple comparisons test was applied for post hoc test. KOA, knee osteoarthritis; NSD1, Nuclear binding SET domain 1.

KOA mouse model was established in this study. Compared with those in the sham group, mice in the KOA group exhibited serious cartilage damage and fibrosis (*p* < 0.01, Figure 1A), elevated OARSI score (*p* < 0.01, Figure 1B), upregulated TNF‐α and IL‐6, downregulated IL‐10 (*p* < 0.01, Figure 1C–E), and poorly expressed NSD1 (*p* < 0.01, Figure 1F,G). To probe the exact role of NSD1 in KOA progression, it was overexpressed in KOA mouse models (*p* < 0.01, Figure 1F,G). Afterward, the KOA + oe‐NSD1 group showed relieved pathological changes in KOA tissues (*p* < 0.01, Figure 1A), degraded OARSI score (*p* < 0.01, Figure 1B), suppressed TNF‐α and IL‐6 levels, and upregulated IL‐10 level (*p* < 0.01, Figure 1C–E). In general, NSD1 overexpression could alleviate cartilage damage in KOA mice.

### 3.2 NSD1 overexpression inhibits ferroptosis in KOA mice

**FIGURE 2:**
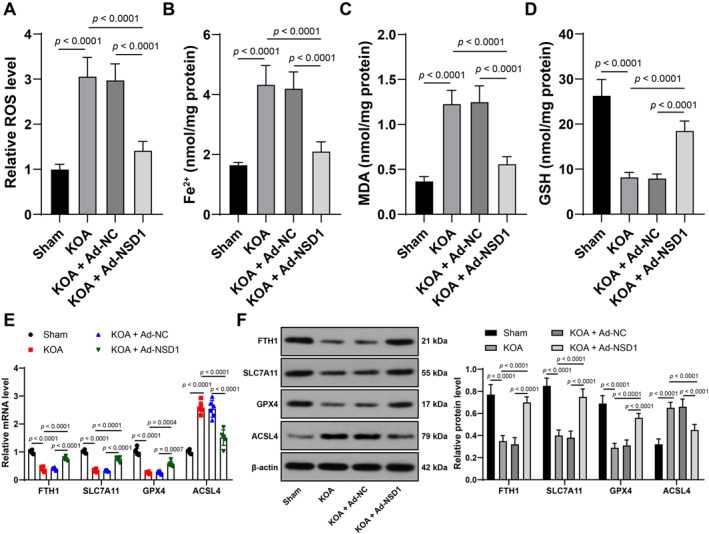
NSD1 overexpression inhibits KOA mouse ferroptosis. Ad‐NSD1 was injected into mice to upregulate NSD1 expression, with Ad‐NC as a control. KOA mouse model was established 24 h after injection and mice were euthanatized, and their knee joints were collected. (A–D) reactive oxygen species (A), Fe^2+^ (B), malonaldehyde (C) and glutathione (D) levels in cartilage tissues. (E, F) ferritin heavy chain 1, solute carrier family 7 member 11, glutathione peroxidase 4 and acyl‐CoA synthetase long‐chain family member 4 levels in cartilage tissues detected by quantitative real‐time polymerase chain reaction (E) and western blot analysis (F). *N* = 6. Data were presented as mean ± standard deviation. One‐way analysis of variance was used to analyze data in panels (A–D). Tukey's multiple comparisons test was applied for post hoc test. KOA, knee osteoarthritis; NSD1, Nuclear binding SET domain 1.

Ferroptosis could promote osteoarthritis progression,^22^ but whether NSD1 influences KOA ferroptosis remains unknown. We analyzed the interaction between NSD1 and ferroptosis in mouse cartilage tissues. The results showed that the KOA group had higher levels of ROS, Fe^2+^ and MDA (*p* < 0.01, Figure 2A–C) but lower levels of GSH, FTH1, SLC7A11 and GPX4 and higher level of ACSL4 (*p* < 0.01, Figure 2D–F) than the sham group; while the KOA + oe‐NSD1 group showed downregulated levels of ROS, Fe^2+^ and MDA and upregulated GSH, FTH1, SLC7A11 and GPX4 and inhibited ACSL4 level (*p* < 0.01, Figure 2A–F), indicating that NSD1 overexpression inhibited ferroptosis in KOA mice.

### 3.3 NSD1 overexpression inhibits IL‐1β‐induced chondrocyte ferroptosis

**FIGURE 3:**
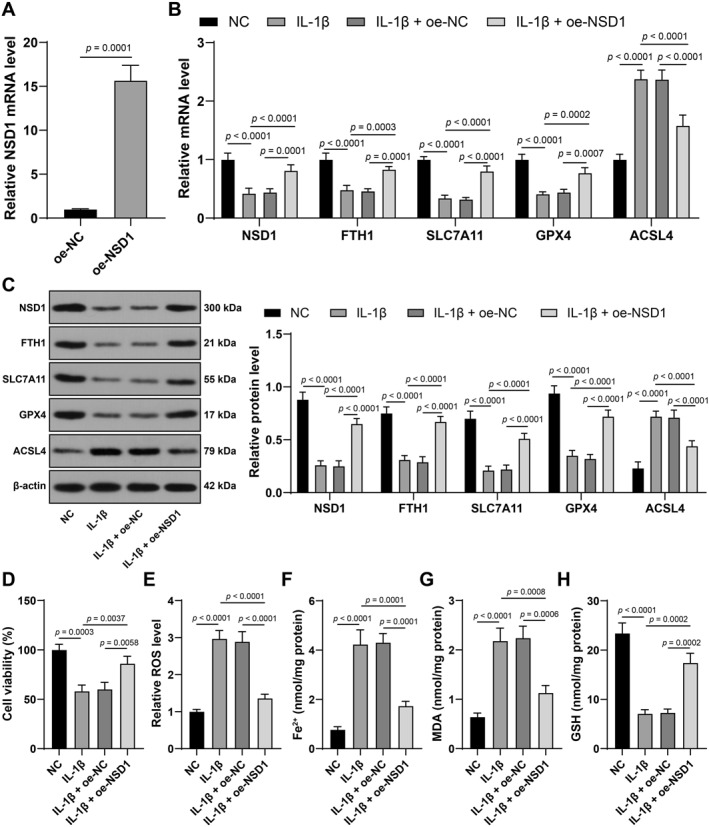
NSD1 overexpression inhibits IL‐1β‐induced chondrocyte ferroptosis. Ad‐NSD1 was injected into mouse primary knee chondrocytes for 48 h to upregulate NSD1 expression, with Ad‐NC as a control. (A) transfection efficiency detected by qRT‐PCR. Afterward, cells were treated with IL‐1β (10 ng/mL) for 24 h to establish knee osteoarthritis cell model, with untreated chondrocytes as the negative control. (B, C) ferritin heavy chain 1, solute carrier family 7 member 11, glutathione peroxidase 4 and acyl‐CoA synthetase long‐chain family member 4 expressions in cartilage tissues detected by qRT‐PCR (B) and western blot analysis (C). (D) cell viability measured by cell counting kit‐8. (E–H) reactive oxygen species (E), Fe^2+^ (F), malonaldehyde (G), and glutathione (H) levels in cartilage tissues. Independent experiments were repeated 3 times. Data were presented as mean ± standard deviation. The *t* test was employed to analyze data in panel (A) two‐way ANOVA was used to analyze data in panels (B, C) and one‐way ANOVA was appointed to analyze data in panels (D, F–H). Tukey's multiple comparisons test was applied for post hoc test. ANOVA, analysis of variance; NSD1, Nuclear binding SET domain 1; qRT‐PCR, quantitative real‐time polymerase chain reaction.

To obtain further knowledge of the effects of NSD1 in vitro, mouse primary knee chondrocytes were separated and cultured with IL‐1β (10 ng/mL) for 24 h to establish in vitro KOA models, in which NSD1 was overexpressed via transfection (*p* < 0.01, Figure 3A–C). IL‐1β treatment downregulated NSD1 expression (*p* < 0.01, Figure 3B,C), discouraged cell viability (*p* < 0.01, Figure 3D), elevated ROS, Fe^2+^ and MDA levels (*p* < 0.01, Figure 3E–G), inactivated levels of GSH, FTH1, SLC7A11 and GPX4 and improved ACSL4 level (*p* < 0.01, Figure 3H,B,C), which were all reversed upon NSD1 overexpression. The above data showed that NSD1 overexpression could inhibit chondrocyte ferroptosis in vitro.

### 3.4 NSD1 enhances H3K36me2 modification to promote KLF9 and ATG14 expression

**FIGURE 4:**
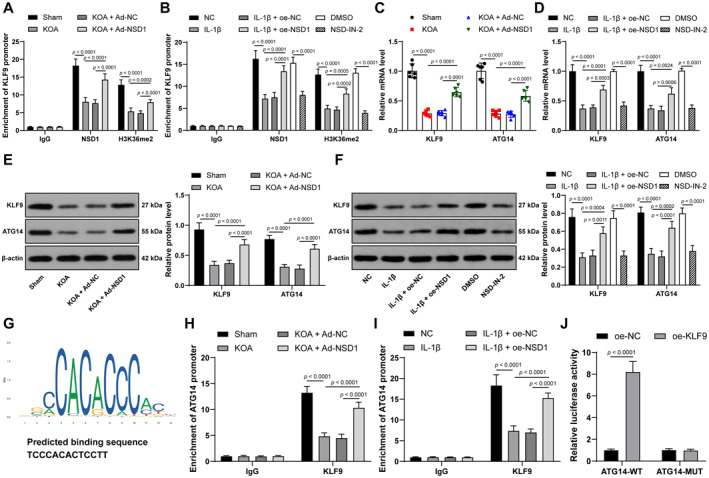
NSD1 enhances H3K36me2 modification to promote KLF9 expression, which further activates ATG14 expression. NSD‐IN‐2 was supplemented into chondrocytes, with the same amount of dimethylsulfoxide as the control. (A, B) the enrichment of NSD1 and H3K36me2 on the KLF9 promoter in cartilage tissues (*n* = 6) and chondrocytes assessed by ChIP assay. (C, D) KLF9 and ATG14 expression in cartilage tissues (*n* = 6) and chondrocytes detected by quantitative real‐time polymerase chain reaction. (E, F) KLF9 and ATG14 expression in cartilage tissues (*n* = 6) and chondrocytes measured by western blot analysis. (G) the binding site of KLF9 on the ATG14 promoter predicted by JASPAR database (https://jaspar.elixir.no/). (H, I) the enrichment of KLF9 on the ATG14 promoter in cartilage tissues (*n* = 6) and chondrocytes assessed by ChIP assay. (J) the binding site between KLF9 and ATG14 promoter verified by dual‐luciferase reporter gene assay. Independent experiments were repeated 3 times. Data were presented as mean ± standard deviation. Two‐way analysis of variance was used to analyze data in panels (A–J). Tukey's multiple comparisons test was applied for post hoc test. ATG14, autophagy‐related 14; ChIP, chromatin immunoprecipitation; KLF9, krüppel‐like factor 9; NSD1, Nuclear binding SET domain 1.

As a crucial histone methyltransferase, NSD1 modulates gene expression via H3K36me2 modification. KLF9 is downregulated in OA patients,^15^, ^23^ so we attempt to elaborate on the specific interaction between NSD1 and KLF9 via H3K36me2 modification. The enrichment of NSD1 and H3K36me2 on the KLF9 promoter was initially assessed by ChIP assay, and it was noticed that the enrichment of NSD1 and H3K36me2 on the KLF9 promoter was reduced in both in vivo and in vitro KOA models, while oe‐NSD1 treatment promoted this enrichment (*p* < 0.01, Figure 4A,B). Likewise, KLF9 was poorly expressed in both in vivo and in vitro KOA models and was abundantly expressed upon oe‐NSD1 treatment (*p* < 0.01, Figure 4C–F). Furthermore, NSD‐IN‐2 was supplemented into chondrocytes, after which it was found that H3K36me2 recruitment on KLF9 promoter was decreased (*p* < 0.01, Figure 4B) and KLF9 expression was downregulated (*p* < 0.01, Figure 4D,F). These findings suggested that NSD1 promoted KLF9 expression via H3K36me2 modification.

KLF9 is capable of modulating its downstream gene expression through transcriptional promotion. ATG14 is inactivated in OA chondrocytes.^19^ The binding sites of KLF9 on the ATG14 promoter were predicted through the JASPAR database (Figure 4G). The binding site between KLF9 and the ATG14 promoter was verified by ChIP assay and dual‐luciferase reporter gene assay (*p* < 0.01, Figure 4H–J). NSD1 overexpression enhanced KLF9 enrichment on the ATG14 promoter (*p* < 0.01, Figure 4J). Furthermore, ATG14 was downregulated in both in vivo and in vitro KOA models and elevated when NSD1 was overexpressed (*p* < 0.01, Figure 4C–F). In summary, KLF9 could transcriptionally promote ATG14 levels by directly binding to the ATG14 promoter.

### 3.5 KLF9 silencing partially counteracts the suppressive effect of NSD1 overexpression on IL‐1β‐induced chondrocyte ferroptosis

**FIGURE 5:**
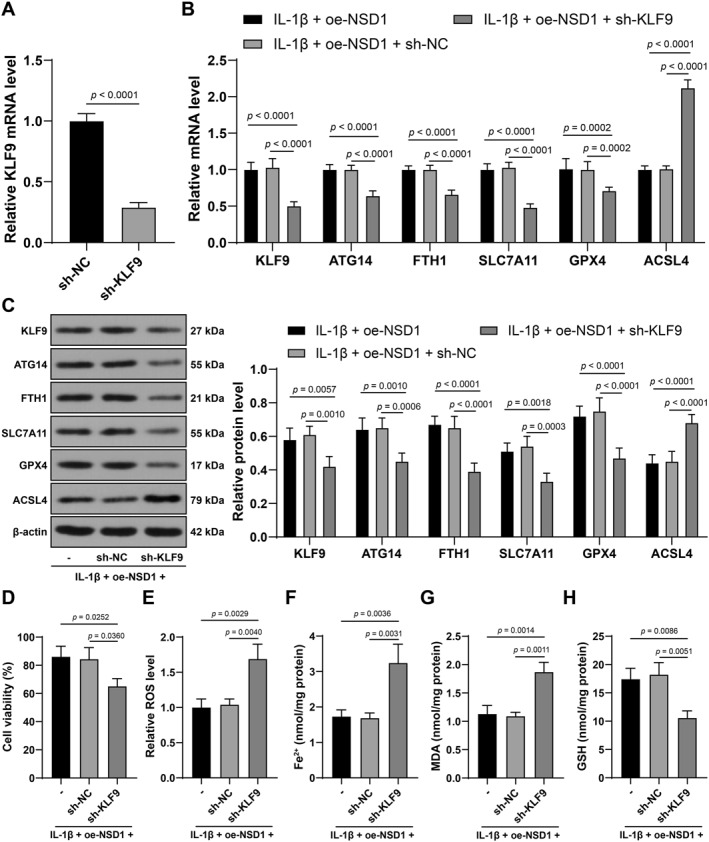
KLF9 silencing partially counteracts the suppressive effect of NSD1 overexpression on IL‐1β‐induced chondrocyte ferroptosis. sh‐KLF9 was transfected into mouse chondrocytes, with sh‐NC as a control, and oe‐NSD1 was also transfected into chondrocytes to perform combination experiment, and cells were treated with IL‐1β (10 ng/mL) for 24 h to establish KOA cell model. (A) transfection efficiency detected by qRT‐PCR. (B, C) KLF9, ATG14, ferritin heavy chain 1, solute carrier family 7 member 11, glutathione peroxidase 4 and acyl‐CoA synthetase long‐chain family member 4 levels in cartilage tissues detected by qRT‐PCR (B) and western blot analysis (C). (D) cell viability measured by cell counting kit‐8. (E–H) reactive oxygen species (E), Fe^2+^ (F), malonaldehyde (G) and glutathione (H) levels in cartilage tissues. Independent experiments were repeated 3 times. Data were presented as mean ± standard deviation. The *t* test was employed to analyze data in panel (A) two‐way ANOVA was used to analyze data in panels (B, C) and one‐way ANOVA was appointed to analyze data in panels (D, F–H). Tukey's multiple comparisons test was applied for post hoc test. ANOVA, analysis of variance; ATG14, autophagy‐related 14; KLF9, krüppel‐like factor 9; NSD1, Nuclear binding SET domain 1; qRT‐PCR, quantitative real‐time polymerase chain reaction. *p < 0.05, **p < 0.01.

sh‐KLF9 was transfected into mouse chondrocytes to suppress KLF9 expression (*p* < 0.01, Figure 5A–C), and oe‐NSD1 was also transfected into chondrocytes for combined experiments. Compared with the IL‐1β + oe‐NSD1 group, the IL‐1β + oe‐NSD1 + sh‐KLF9 group showed quenched cell viability (*p* < 0.05, Figure 5D), elevated ROS, Fe^2+^ and MDA levels (*p* < 0.01, Figure 5E–G), downregulated levels of GSH, ATG14, FTH1, SLC7A11, and GPX4 and elevated ACSL4 expression (*p* < 0.01, Figure 5B,C,H), suggesting that KLF9 exhaustion partially counteracts the suppressive effect of NSD1 overexpression on IL‐1β‐induced chondrocyte ferroptosis.

### 3.6 ATG14 knockdown partially reverses the suppressive effect of NSD1 overexpression on IL‐1β‐induced chondrocyte ferroptosis

**FIGURE 6:**
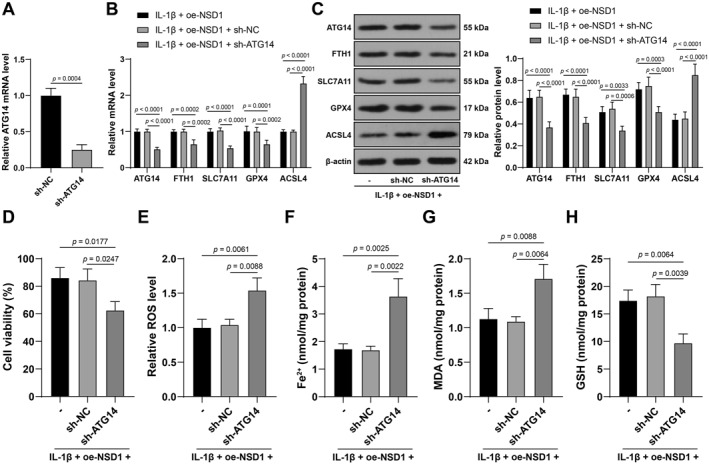
ATG14 knockdown partially reverses the suppressive effect of NSD1 overexpression on IL‐1β‐induced chondrocyte ferroptosis. sh‐ATG14 was transfected into mouse chondrocytes, with sh‐NC as a control, and oe‐NSD1 was also transfected into chondrocytes to perform combination experiment, and cells were treated with IL‐1β (10 ng/mL) for 24 h to establish knee osteoarthritis cell models. A, transfection efficiency detected by qRT‐PCR. B, C, ATG14, ferritin heavy chain 1, solute carrier family 7 member 11, glutathione peroxidase 4 and acyl‐CoA synthetase long‐chain family member 4 expressions in cartilage tissues detected by qRT‐PCR (B) and western blot analysis (C). (D) cell viability measured by cell counting kit‐8. (E–H) reactive oxygen species (E), Fe^2+^ (F), malonaldehyde (G) and glutathione (H) levels in cartilage tissues. Independent experiments were repeated 3 times. Data were presented as mean ± standard deviation. The *t* test was employed to analyze data in panel A, two‐way ANOVA was used to analyze data in panels (B and C) and one‐way ANOVA was appointed to analyze the data in panels (D, F–H). Tukey's multiple comparisons test was applied for post hoc test. ANOVA, analysis of variance; ATG14, autophagy‐related 14; NSD1, Nuclear binding SET domain 1; qRT‐PCR, quantitative real‐time polymerase chain reaction.

sh‐ATG14 was transfected into mouse chondrocytes to suppress ATG14 expression (*p* < 0.01, Figure 6A–C), and oe‐NSD1 was also transfected into chondrocytes for combined experiments. Compared with the IL‐1β + oe‐NSD1 group, the IL‐1β + oe‐NSD1 + sh‐ATG14 group showed quenched cell viability (*p* < 0.05, Figure 6D), promoted ROS, Fe^2+^ and MDA levels (*p* < 0.01, Figure 6E–G, 6B–C) and downregulated levels of GSH, FTH1, SLC7A11, and GPX4 (*p* < 0.01, Figure 6H, 6B–C), suggesting that ATG14 knockdown partially reverses the suppressive effect of NSD1 overexpression on IL‐1β‐induced chondrocyte ferroptosis.

## 4 DISCUSSION

**FIGURE 7:**
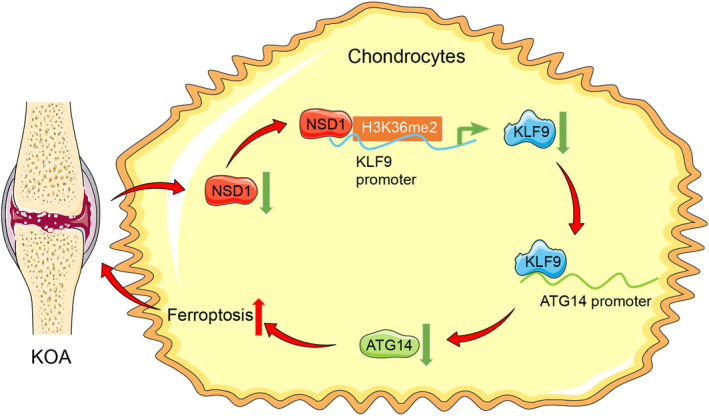
Nuclear binding SET domain 1 is poorly expressed in knee osteoarthritis and decreases KLF9 promoter H3K36me2 modification to inhibit KLF9 expression, thereby suppressing the transcriptional promotion effect of KLF9 on ATG14 to downregulate ATG14 expression, so as to enhance chondrocyte ferroptosis. ATG14, autophagy‐related 14; KLF9, krüppel‐like factor 9.

KOA represents a common and severe joint disease affecting a huge number of people attributed to growing obesity and aging population, resulting in high disability rates.^24^ Ferroptosis is a kind of complex iron‐related cell death that mediates inflammation and differentiation activities via molecular pathways and alters KOA homeostasis, eventually aggravating cartilage dysfunction and joint pain.^25^ Histone modification acts as a key mechanism in OA by controlling different cellular biological activities, including cell integrity, renewal, death, apoptosis, and ferroptosis.^26^ H3K36 methyltransferase NSD1 is a popular candidate in novel epigenetic treatment for human disorders for its prominent role in targeting H3K36me2 modification and the subsequent regulation of gene transcription and cellular interactions.^27^ In the current research, we explored the intersection between NSD1 and chondrocyte ferroptosis with the involvement of the KLF9/ATG14 pathway via H3K36me2 modification (Figure 7).

Abnormal inflammatory cytokine release is a major feature of KOA.^28^ NSD1 is downregulated in an inflammatory environment, and it is associated with impaired immune function and disrupted metabolic development.^29^ Furthermore, NSD1 expression was inactivated in KOA, while NSD1 overexpression regulated H3K36me2 recruitment to improve chondrocyte activity and suppressed cellular failure.^11^ NSD1 Likewise, in our study, NSD1 was poorly expressed in KOA models, and when it was overexpressed, TNF‐α and IL‐6 levels were suppressed and IL‐10 level was upregulated. Reduced TNF‐α and IL‐6 levels are linked to restored cartilage tissue function and relieved KOA.^30^ Significantly, under‐expressed NSD1 accelerated OA severity and influenced cartilage growth, differentiation, and injury repair.^31^ In OA subjects, NSD1 expression was quenched, and cartilage injury was aggravated with the involvement of inhibited chondrogenic differentiation and impaired cartilage homeostasis.^12^ In general, NSD1 overexpression could alleviate inflammatory responses induced by KOA. On the other hand, as a kind of cell death related to lipid peroxidation, ferroptosis plays a significant role in OA progression.^32^ Our results showed that levels of ROS, Fe^2+^ and MDA were downregulated while GSH, FTH1, SLC7A11, and GPX4 levels were upregulated in KOA mice and IL‐1β‐induced chondrocyte after NSD1 overexpression. Besides, when GSH and SLC7A11 were downregulated and MDA expression was encouraged, chondrocyte function was impaired, ferroptosis was catalyzed and oxidative damage was exacerbated in OA.^33^ A recent study has pointed out that the protective effect of NSD1 upregulation could promote chondrocyte viability and inhibit ferroptosis in IL‐1β‐stimulated chondrocytes and KOA mice.^11^ These findings showed that NSD1 overexpression inhibited ferroptosis in KOA mice.

A prominent mechanism of NSD1 in different types of diseases is to control its downstream genes via transcriptional modulation of H3K36me2.^34^ In our research, the enrichment of NSD1 and H3K36me2 was reduced on the KLF9 promoter in OA models, which inspired us to elaborate the network between KLF9 and KOA. KLF9 expression is discouraged in joint disorders, and it serves as a responsible diagnostic biomarker for OA,^35^ illustrating that KLF9 could be a valuable target in KOA. To further illustrate the effect of KLF9 on KOA, we downregulated KLF9 in IL‐1β‐stimulated chondrocytes through shRNA transfection and found that ROS, Fe^2+^ and MDA levels were improved and GSH, ATG14, FTH1, SLC7A11, GPX4 levels were downregulated while ACSL4 level was upregulated. KLF9 silencing increased ROS and MDA levels in rheumatoid arthritis.^36^ Collectively, KLF9 blocked chondrocyte ferroptosis in KOA.

KLF9 could mitigate inflammatory responses and cartilage damage in rheumatoid arthritis via the transcriptional regulation of its downstream gene.^37^ Furthermore, our data demonstrated the binding site between KLF9 and ATG14. Although there is no direct report about the relation between KLF9 and ATG14, a previous document has pointed out that KLF5 was positively related to ATG expression, so as to strengthen multiple organ system function.^16^ According to Wang et al., effective drug administration facilitated chondrocyte repair and renewal and abolished joint damage in rheumatoid arthritis by increasing ATG14 expression.^38^ ATG14 contributed to active chondrocyte differentiation and reduced oxidative injury to block osteoporosis.^39^ To figure out the crosstalk between ATG14 and KOA, sh‐ATG14 was transfected into mouse chondrocytes to suppress ATG14 expression, and the results exhibited that ROS, Fe^2+^ and MDA levels were promoted and levels of GSH, FTH1, SLC7A11, and GPX4 were inactivated, and ACSL4 level was activated, suggesting that ATG14 could ameliorate chondrocyte ferroptosis. Altogether, ATG14 helped to prevent KOA chondrocyte ferroptosis.

To sum up, our results supported that NSD1 enhanced KLF9 expression to improve ATG14 expression via H3K36me2 modification, thus relieving KOA cartilage ferroptosis. These results are prospective in promoting future therapy of KOA. Nevertheless, there are several limitations. We only investigated ferroptosis in chondrocytes but not in other cells such as osteoblasts and osteoclasts. Besides, NSD1, as an important H3K36me2 methyltransferase, might mediate the expression of other genes via histone modification. Moreover, it remains to be further explored whether NSD1 is involved in other forms of cell death in KOA progression. The above‐mentioned mechanisms could be further discussed in future research.

## AUTHOR CONTRIBUTIONS

**Qinglei Yang:** Conceptualization; data curation; investigation; visualization; writing—original draft; writing—review and editing. **Rugang Li:** Conceptualization; data curation; methodology; visualization. **Zhiqiang Hu:** Data curation; methodology; validation. **Wengang Zhu:** Formal analysis; investigation; validation. **Hongying Yu:** Conceptualization; methodology; supervision; writing—review and editing.

## CONFLICT OF INTEREST STATEMENT

The authors declare no conflicts of interest.

## ETHICS STATEMENT

The protocol was also approved by the Institutional Animal Care and Use Committee of Yuebei People's Hospital Affiliated to Shantou University Medical College. Significant efforts were made to reduce the animal number and suffering.

## Data Availability

Data will be made available on request.
